# Gould’s Pocket Pronouncing Dictionary

**Published:** 1898-12

**Authors:** 


GOULD'S POCKET PRONOUNCING DICTIONARY. By George M. Gould. P. Blakiston's Son & Co., Philadelphia.

The new edition of this little book comes to us much improved. It is little in size only. The number of words has been almost doubled, and the old words, as well as the new, have been carefully edited.

We all know how limited is the usefulness of some of the dictionaries to a student wishing a clear, concise definition. One is referred from place to place and word to word, in the end having a very cloudy idea or understanding of the meaning of the word in question. Many instances of this inefficient editing could be cited, which no doubt have caused much annoyance to students, teachers, and writers. This fault is notably absent in the Gould dictionaries. The results of Dr. Gould's work in dictionary making is a decided achievement, and there is probably no one to-day better equipped for such work.

In this work, so small that it can be carried conveniently in the pocket, there are twenty-one thousand useful words, with correct pronunciation and clear definition. Many of the newer chemical terms, also, have their formula given, and there are numerous valuable tables introduced. It is truly "infinite riches in little room."

